# Modeling historical tuberculosis epidemics among Canadian First Nations: effects of malnutrition and genetic variation

**DOI:** 10.7717/peerj.1237

**Published:** 2015-09-24

**Authors:** Sarah F. Ackley, Fengchen Liu, Travis C. Porco, Caitlin S. Pepperell

**Affiliations:** 1 Department of Epidemiology and Biostatistics, University of California San Francisco, San Francisco, CA, USA; 2 Proctor Foundation, University of California San Francisco, San Francisco, CA, USA; 3 Departments of Medicine (Infectious Diseases) and Medical Microbiology and Immunology, University of Wisconsin Madison, Madison, WI, USA

**Keywords:** Tuberculosis, Mathematical model, First Nations, Malnutrition, Genetic predisposition to disease, Epidemics

## Abstract

Late 19th century epidemics of tuberculosis (TB) in Western Canadian First Nations resulted in peak TB mortality rates more than six times the highest rates recorded in Europe. Using a mathematical modeling approach and historical TB mortality time series, we investigate potential causes of high TB mortality and rapid epidemic decline in First Nations from 1885 to 1940. We explore two potential causes of dramatic epidemic dynamics observed in this setting: first, we explore effects of famine prior to 1900 on both TB and population dynamics. Malnutrition is recognized as an individual-level risk factor for TB progression and mortality; its population-level effects on TB epidemics have not been explored previously. Second, we explore effects of heterogeneity in susceptibility to TB in two ways: modeling heterogeneity in susceptibility to infection, and heterogeneity in risk of developing disease once infected. Our results indicate that models lacking famine-related changes in TB parameters or heterogeneity result in an implausibly poor fit to both the TB mortality time series and census data; the inclusion of these features allows for the characteristic decline and rise in population observed in First Nations during this time period and confers improved fits to TB mortality data.

## Introduction

In the late 19th and early 20th centuries, large-scale epidemics of tuberculosis (TB) swept through First Nations communities in Western Canada (Ferguson, 1928; Lux, 2001; Daschuk & Hackett, 2006; Pepperell et al., 2010). TB epidemics in these communities resulted in mortality rates six times higher than the highest recorded TB mortality rates in Europe (Grigg, 1958), and were followed by a rapid decline in mortality following the epidemic peak (Ferguson, 1928).

Mathematical models have been fit to TB mortality time series data from other settings (Murray & Salomon, 1998; Dye et al., 1998; Vynnycky & Fine, 1999). Historical data from the late 19th and early 20th centuries from First Nations communities have not, however, been extensively explored using modeling techniques (Clark & Cameron, 2009). Compartmental models have been used previously to estimate and predict future trends in annual risk of infection with data dating back to 1926 (Clark & Vynnycky, 2004; Clark & Cameron, 2009). Data from these epidemics provide a unique opportunity to observe TB dynamics in the early years of an epidemic: while TB mortality time series data were not collected until about 100 years after the start of the epidemics in Europe, data from First Nations communities are available within one to two decades of the epidemic start times as determined by historical accounts (Daschuk & Hackett, 2006).

It has been proposed that malnutrition (Daschuk & Hackett, 2006) and crowding (Ferguson, 1928; Lux, 2001) played major roles in creating and maintaining high TB mortalities in First Nations communities during this time period. Early observers also speculated about the role the TB epidemic played in altering the composition of these populations, by increasing relative proportions of individuals with intrinsic resistance to the disease (Ferguson, 1928).

Some aspects of secular TB trends in other settings are unexplained and have attracted research interest. For example, TB mortality declined in North America and Europe prior to the availability of effective chemotherapy (McKeown & Record, 1962; Vynnycky & Fine, 1999). Possible explanations for the decline in TB mortality around the turn of the 20th century in Europe include public health interventions and improvements in housing and nutrition (McKeown & Record, 1962; Szreter, 1988; Mitchell, 1992). Models that account for historical changes in crowding can successfully explain the historical decline in Europe (Vynnycky & Fine, 1999). However, unlike Europe over the course of the 20th century, housing densities and institutionalization of First Nations communities increased as the late 19th century TB epidemic took off (Milloy, 1999; Lux, 2001). Crowding is in fact still a problem in First Nations communities (Clark, Riben & Nowgesic, 2002; Larcombe et al., 2011). It is therefore unlikely that the sharp decline in TB mortality observed in the early 20th century can be explained by improving living conditions.

Building upon previously published TB models (ReVelle, Lynn & Feldmann, 1967; Blower et al., 1995; Dye et al., 1998; Vynnycky & Fine, 1999), we simulate TB epidemics in Western Canadian First Nations at the turn of the 20th century. Based on the history of First Nations peoples in this region, we propose additional model features not present in previously published TB models: first, we propose that severe famine in the late nineteenth century may have led to time-varying model parameters as the severity of the famine eased in the 20th century (Lux, 2001; Daschuk & Hackett, 2006). Specifically, we allow the following model parameters to be time varying: TB mortality rates, TB progression rates, protective immunity to TB, as well as birth and non-TB death rates.

Second, and in addition to exploring models with time-varying parameters, we propose two alternative TB models in which we model genetic heterogeneity in TB susceptibility. In the first model—the innate immunity model—a fraction of the starting population has increased susceptibility to infection with *Mycobacterium tuberculosis* (*M. tuberculosis*). Over the course of the epidemic, this fraction decreases, as individuals with greater susceptibility are less likely to produce offspring. In the second model—the adaptive immunity model—a fraction of the starting population has a higher risk of TB progression following infection with *M. tuberculosis*. Again, over the course of the epidemic, this fraction decreases, as individuals with greater susceptibility are less likely to produce offspring.

Using historical mortality data from 1885 to 1940 and census data from 1883 to 1939 from First Nations communities in Saskatchewan, Canada, we compare TB models with time-varying parameters and models with genetic heterogeneity to a “classical” TB model, which does not include these features. Fitting models to the rarely observed takeoff point of severe TB epidemics presents a unique opportunity to gain insight into fundamental features of TB transmission dynamics in this and other settings. TB is a global public health emergency, and it remains a significant challenge to the health and well-being of Canadian First Nations (Canadian Thoracic Society and the Public Health Agency of Canada, 2013). A better understanding of epidemic dynamics is needed to address this challenge to global public health.

## Materials and Methods

### Data

Data analyzed here were completely de-identified (no geographic identifiers below the level of province) and aggregated prior to the study. The original data sources are described in (Pepperell et al., 2010). We included TB mortality data from 7 First Nations communities in Saskatchewan, Canada. Data were available from 3 communities between 1885 and 1940, and from 4 communities between 1897 and 1940. The data from 1885 to 1940 were aggregated into one group and the data from 1897 to 1940 were aggregated into a second group. Within these groupings, communities were in close proximity and with similar dynamics, as described elsewhere (Pepperell et al., 2010). Canadian Census reports and Indian Affairs Annual Reports of census were included for years from 1883 to 1940 for which community level data were available for all reserves in a cluster (Library and Archives Canada, 2014). Library and Archives Canada (2014) provided contemporary birth rate data for these and similar communities in the area (Lux, 2001).

### Natural history of TB

We briefly review relevant features of the natural history and epidemiology of tuberculosis. TB is largely transmitted by aerosols produced from coughing in individuals with active pulmonary disease. The probability of transmission within a short time frame is low (Baxter, 1993), and most infections occur after repeated exposure to an infectious individual (Baltimore, 1993). Following infection with *M. tuberculosis*, 5 to 10% of individuals go on to develop active TB within a short timeframe of one year (Styblo, 1986). The majority of infected individuals, however, remain latently infected and asymptomatic, with a 5 to 10% risk of developing active disease over their lifetime (HL & SD, 1988; Blower et al., 1995). Of individuals who develop active disease, a fraction develops smear-positive disease, which accounts for the majority of transmission; the remaining individuals develop smear-negative disease, which includes less-infectious forms of pulmonary disease as well as extra-pulmonary disease (Narain, 1980). Slightly more than half of individuals develop smear-positive (infectious) disease (Blower et al., 1995; Aparicio & Castillo-Chavez, 2009). A small fraction of active cases recover naturally; however, these individuals can relapse due to reactivation (Tiemersma et al., 2011). In the absence of treatment, the average duration of TB from onset to death or self-cure is about 3 years (Tiemersma et al., 2011).

**Figure 1:**
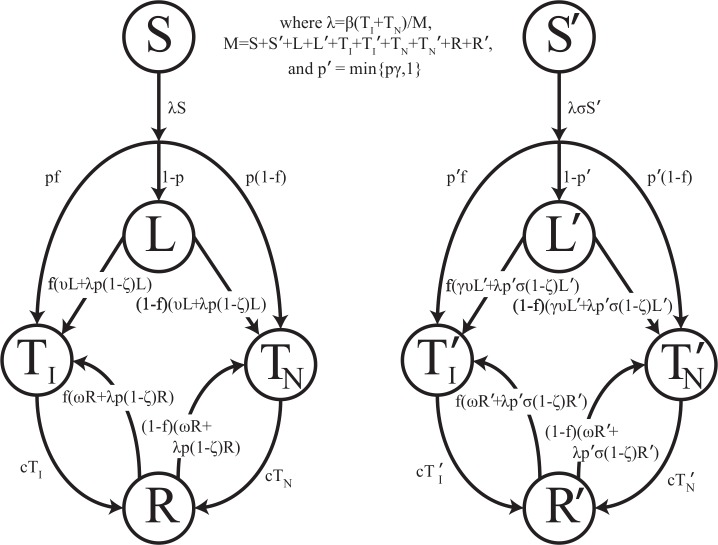
Transmission diagram. Representation of compartmental TB models 0, 1, 2, and 3. Individuals who are more genetically susceptible are in the primed states, whereas less genetically susceptible individuals are in the unprimed states. *S*, *L*, *T_I_*, *T_N_*, and *R* represent the numbers of people in the susceptible, latent, infectious active disease, non-infectious active disease, and recovered groups, respectively, as a function of time for the less susceptible group. *S*′, *L*′, ${T}_{I}^{{\prime}}$, ${T}_{N}^{{\prime}}$, and *R*′ represent the numbers of people in the susceptible, latent, infectious active disease, non-infectious active disease, and recovered groups, respectively, as a function of time for the more susceptible group. Model parameters are given in Table 1. For models 0 and 1, no individuals start out in *S*′, and thus all primed states remain empty. For models 2 and 3, a fraction of individuals start in *S*′. For model 2, *σ* ≠ 0 and *y* = 0. For model 3, *σ* = 0 and *y* ≠ 0. For simplicity, background mortality and TB specific mortality were omitted from the diagram.

Mathematically, we represent the course of TB according to Fig. 1. We distinguish the following non-TB state and TB states: (1) uninfected individuals, (2) latently-infected individuals who have been infected with TB but are asymptomatic and non-infectious, (3) infectious active disease (pulmonary, smear-positive) in which individuals are symptomatic and at a greatly increased risk of death due to disease, (4) non-infectious active disease (pulmonary but smear-negative or extra-pulmonary) in which individuals are symptomatic and at an increased risk of death due to disease, and (5) individuals who recover naturally from active disease and may progress to active disease again. Unlike other TB models, no states involving treatment or recovery following chemotherapy are necessary since there was no chemotherapy available prior to the demonstration of efficacy of streptomycin against tuberculosis during the mid-1940s (Youmans & McCarter, 2011; Hinshaw, Feldman & Pfuetze, 1946).

It has been suggested that the sharp TB dynamics observed in First Nations reserves around 1900 are due to heterogeneity in susceptibility that existed at the initiation of the epidemic: that is, mortalities declined as the proportion of the population composed of highly susceptible individuals shrank (Ferguson, 1928; Lux, 2001). In TB endemic settings, the disease has its severest impacts on very young children and individuals of childbearing age (Wood et al., 2011). This suggests that TB has the potential to impose strong selection on human evolution. There is evidence to suggest that people vary in their susceptibility to TB, but the mechanism by which some individuals are more susceptible remains unclear (Abel et al., 2014). Heterogeneity in susceptibility to *M. tuberculosis* infection (innate immunity model) and heterogeneity in risk of progression once infected (adaptive immunity model) are both possible mechanisms rendering some people more susceptible to TB (Abel et al., 2014). We represent both of these possibilities by including two groups of individuals, as shown in Fig. 1, where individuals in the primed states are more susceptible than individuals in the unprimed states.

In previous genetic models of TB susceptibility, it was assumed that ethnic risk groups differed in the length of exposure to TB, and, as a result of long-term selection for genetic variants conferring resistance, differed in their susceptibility to *M. tuberculosis* infection and disease (Stead et al., 1990). We are instead hypothesizing that ecological drivers of epidemic TB, such as famine and crowding, resulted in strong selection on existing genetic variation within First Nations.

It has been argued previously that First Nations experienced such severe TB epidemics because they were naïve to *M. tuberculosis* (Motulsky, 1989). We do not consider this a plausible explanation for the observed dynamics because First Nations were not naïve populations: pathogen genetic data indicates that Western Canadian First Nations were exposed to *M. tuberculosis* during contact with European immigrants in the 18th century, one hundred years prior to the onset of epidemic TB (Pepperell et al., 2011a). Prior to the severe 19th century epidemics driven by famine and crowding, TB evidently persisted in these populations in a mild endemic form that is likely to have been associated with weak selection against susceptibility variants. There is also evidence of TB throughout Native American populations prior to contact with European immigrants, which raises the possibility that Western Canadian populations encountered *M. tuberculosis* prior to their exposure during the fur trade (Stone et al., 2009).

We refer to the epidemic start time as the time at which TB epidemics took off in the late 19th century and not the time at which *M. tuberculosis* was introduced by European immigrants. The genetic evidence indicates that dispersal occurred prior to TB epidemics, which were later spurred by famine and crowding beginning in the late 19th century (Pepperell et al., 2011a). It is for this reason that genetic selection, if it occurred, would not have occurred until the late 19th century when nutrition was poor, TB disease was widespread, and living conditions were crowded.

### Time varying parameters

Starting in the 1870s, First Nations people in Saskatchewan underwent a large-scale transition from nomadic or semi-nomadic lifestyles to settlement on reserves with attempts to take up farming; this transition was associated with severe famine (Lux, 2001). To account for this, we included five famine-related parameters in one of our models. It is well known that malnutrition, as well as deficiencies of specific micronutrients (Getz, Long & Henderson, 1951; Taylor & Camargo, 2011; Cegielski, Arab & Cornoni-Huntley, 2012), affect the risk of active disease and death among those with active disease. Malnutrition has been conceived of as an individual-level risk factor. Given the exceptionally high rates of TB observed in First Nations reserves coincident with profound famine, we sought to explore potential effects of malnutrition on dynamics of TB. We included five TB parameters that are plausibly affected by malnutrition: the TB death rate (Rao et al., 1998; Gustafson et al., 2007), the probability of fast progression (Downes, 1950; Cegielski, Arab & Cornoni-Huntley, 2012), the level of protective immunity to reinfection conferred by latency, and the rate of progression from latency (Cegielski, Arab & Cornoni-Huntley, 2012). There is reason to believe that malnutrition does not affect susceptibility to initial infection (Cegielski, Arab & Cornoni-Huntley, 2012), so this was not included as a famine-related parameter. Additionally, two demographic parameters are plausibly affected by malnutrition: the birth (Salam, Das & Bhutta, 2014) and death rates (Bresnahan & Tanumihardjo, 2014). Since famine-related changes in parameters are not well characterized, they were allowed to vary by up to a factor of 3. The late 19th century famine contributed to a demographic crash in Saskatchewan Plains First Nations, with some recovery of population numbers evident by the turn of the 20th century (Lux, 2001). The demographic recovery could have been due to both direct effects of the famine easing, as well as indirect effects of improving nutrition on susceptibility to TB (Lux, 2001).

In addition to its association with famine and malnutrition in the late 19th century, the transition from nomadic or semi-nomadic lifestyles to life on a government reserve also resulted in increased population densities (Lux, 2001). The late 19th and early 20th centuries were marked by increasing institutionalization of First Nations, with attendant negative impacts on their health (Milloy, 1999; Lux, 2001; Waldram, Herring & Young, 2006). This is in sharp contrast to Europe at the same time where living conditions were improving (McKeown & Record, 1962; Vynnycky & Fine, 1999). We account for this increase in crowding by allowing the effective contact rate to increase by a constant percentage between 0% and 2% per year over this period.

### Models

We simulated the transmission and progression of TB deterministically using the differential equations outlined in the supplement using a time step of one month. The simulation outputted the number of individuals who were susceptible, latent, infectious active cases, non-infectious active cases, and recovered as well as the cumulative number of mortalities at each time step. We evaluated the evidence for four different TB models, comparing models 1, 2, and 3 to the baseline model (model 0):

0. Baseline Model, which models the progression of TB as outlined above and depicted in Fig. 1 without any time-varying famine-related parameters or heterogeneity in susceptibility. This model will be used as comparison for the subsequent models.
1. Famine model, which is the same as the baseline model but allows for time-varying famine-related parameters, which change according to a step-function around 1900 (between 1895 and 1905), around when population levels began to increase (Lux, 2001). The time varying parameters are outlined in Table 1.
2. Innate Immunity Model, which is the same as the baseline model with the addition of a group of individuals who are more susceptible to infection with *M. tuberculosis*. Over the course of the epidemic, this group decreases in size, as individuals with greater genetic susceptibility are less likely to produce offspring.
3. Adaptive Immunity Model, which is the same as the baseline model with the addition of a group of individuals who are at higher risk of progression to active disease once infected. Over the course of the epidemic, this group decreases in size, as individuals with greater risk of disease are less likely to produce offspring.

**Table 1:** Parameters and associated ranges.

Parameter name	Description	Minimum	Maximum	References
*β*	Effective contact rate, average number of cases caused by one case in a fully susceptible population (per year per infectious case)^a^	2	20	(Blower et al., 1995; Aparicio & Castillo-Chavez, 2009)
*p*	Proportion of new infections that develop disease within a year, for susceptible and latent individuals (post-famine)^b^	0	0.30	(Blower et al., 1995; Gomes et al., 2004).
*f*	Probability of developing infectious TB for individuals who develop active disease	0.5	0.85	(Blower et al., 1995; Aparicio & Castillo-Chavez, 2009)
*ζ*	Fractional protection conferred by latent infection to reinfection (post-famine)^b^	0.0	0.8	(Ferguson, 1928; Gomes et al., 2004)
*ν*	Rate of progression from latent to active disease (post-famine) (per year)^b^	0.00256	0.00527	(Blower et al., 1995; Gomes et al., 2004)
*μ*	Background mortality rate (post-famine) (per year)^b^	0.015	0.03	(Blower et al., 1995)
*μ*_TB_	TB mortality rate (post-famine) (per year)^b^	0.2	0.7	(Blower et al., 1995; Gomes et al., 2004; Tiemersma et al., 2011)
*c*	Self-cure rate (per year)	0.021	0.086	(Blower et al., 1995; Tiemersma et al., 2011)
*ω*	Relapse rate (per year)	0.01	0.03	(Blower et al., 1995)
*σ*	Factor by which susceptibility to infection among more susceptible individuals is increased	1	1,000	
*γ*	Factor by which risk of progression among more susceptible individuals is increased	1	1,000	
*1-s*	Fraction of individuals with increased susceptibility to TB infection at the start of the epidemic or with increased risk of progression at the start of the epidemic	0	1	
Λ	Birth rate (post-famine) (births per 1,000 population per year)	30	50	(Lux, 2001)
*δ_p_*	Factor by which famine conditions increased *p*	1	3	
*δ*_*μ*_TB__	Factor by which famine conditions increased *μ*_TB_	1	3	
*δ*_*μ*_	Factor by which famine conditions increased *μ*	1	3	
*δ*_*ν*_	Factor by which famine conditions increased *ν*	1	3	
*δ*_*ζ*_	Factor by which famine conditions decreased *ζ*	0.3	1	
*δ*_*β*_	Percent increase in *β* per year following 1880	0%	2%	(Milloy, 1999; Lux, 2001)
*δ*_Λ_	Factor by which famine conditions decreased the birth rate	0.3	1	
*t*_1_	Start time of epidemic for cluster 1	1870	1883	(Lux, 2001), census data
*t*_2_	Start time of epidemic for cluster 2	1870	1883	(Lux, 2001), census data
*p*_1_	Starting population of cluster 1	800	1,600	Census data
*p*_2_	Starting population of cluster 2	400	1,200	Census data
*ϵ*	Time when famine conditions improved	1895	1905	(Lux, 2001), census data

**Notes.**

a See associated *δ* parameter since this quantity described by this parameter changes over time.

b See associated *δ* parameter and *ϵ* since the quantity described by this parameter changes pre-/post- famine.

### Parameters

Model parameters with descriptions are given in Table 1, along with a plausible range for each parameter. Ranges were determined from the epidemiologic and modeling literature.

### Analysis

All analysis was performed in R version 3.1.0. The following procedure was used to reduce the parameter space: For the full model, the model in which models 0–3 are nested, containing all 25 parameters listed in Table 2, we selected Latin Hypercube samples of parameters chosen uniformly from the parameter ranges given in Table 1. A total of 128 Latin Hypercube samples of 1,024 parameter sets were used, for a total of 131,072 parameters sets.

**Table 2:** Parameter estimates. Parameter estimates for the baseline model, famine model, intrinsic immunity model, and adaptive immunity model, respectively, to two significant figures (or the nearest year). Discrepancies are given to the nearest tenth. Best parameter estimates were obtained from optimization. Intervals were estimated from the 2.5%ile and 97.5%ile of the fits to the bootstrap data sets.

Parameter	Description	Model 0	Model 1	Model 2	Model 3
		Best estimate (interval)	Best estimate (interval)	Best estimate (interval)	Best estimate (interval)
*p*	Proportion of new infections or new reinfections that develop disease within a year, for susceptible, latent, and recovered individuals (post-famine)	0.30 (0.30, 0.30)	0.26 (0.19, 0.27)	0.042 (0.028, 0.054)	0.18 (0.10, 0.23)
*β*	Effective contact rate, average number of cases caused by one case in a fully susceptible population (per year per infectious case)	10 (9.5, 18)	3.4 (2.0, 4.1)	2.7 (2.2, 4.1)	2.43 (2.2, 3.2)
*s*	Fraction of individuals with normal (lower) susceptibility to TB infection at the start of the epidemic	–	–	0.20 (0.18, 0.26)	0.40 (0.30, 0.50)
*t*_1_	Start time of epidemic for cluster 1	1870 (1870, 1875)	1872 (1870, 1875)	1873 (1872, 1877)	1873 (1871, 1878)
*δ_p_*	Factor by which famine conditions increased *p*	–	2.3 (2.3, 3.0)	–	–
*t*_2_	Start time of epidemic for cluster 2	1870 (1870, 1870)	1872 (1870, 1872)	1872 (1870, 1875)	1872 (1870, 1876)
*ζ*	Fractional protection conferred by latent infection to reinfection (post-famine)	0.80 (0.80, 0.80)	0.80 (0.78, 0.80)	0.74 (0.67, 0.78)	0.51 (0.00, 0.78)
*f*	Probability of developing infectious TB for individuals who develop active disease	0.85 (0.50, 0.85)	0.56 (0.50, 0.85)	–^a^	–^a^
*μ*_TB_	TB mortality rate (post-famine) (per year)	0.70 (0.59, 0.70)	–^a^	–^a^	–^a^
*σ*	Relative susceptibility to infection among more susceptible individuals compared with normally susceptible individuals	–	–	46 (32, 71)	–
*γ*	Factor by which risk of progression among more susceptible individuals is increased	–	–	–	5.43 (4.42, 9.23)
	Lowest discrepancy	186.7 (136.5, 241.5)	65.1 (45.5, 81.4)	62.4 (44.7, 75.8)	66.9 (46.4, 85.0)

**Notes.**

a For each model, the seven most important parameters relevant to that model were fit to the data. For models 2 and 3, *f* and *μ*_TB_ were dropped in order to include *s* and *σ* and *s* and *γ*, respectively. For model 1, *μ*_TB_ was dropped in order to include *δ_p_*.

For each parameter set, a discrepancy was calculated on the relative scale (see equation in the Supplemental Information). The total population and total TB mortality discrepancies were weighted equally. In the Supplemental Information, we show that fits on the absolute scale yield similar results. We used the relative scale for our main analysis since the method to obtain confidence intervals, described below, would not work on the absolute scale, as we would obtain mortalities less than zero. That is, on the absolute scale, a large negative absolute discrepancy added to a small mortality would give a negative bootstrapped mortality. Using the relative scale avoids this issue. Using these results, we fit the discrepancies to the parameters using the polynomial spline-fitting algorithm *polymars* in the *polspline* package in R to determine the seven most influential parameters for each of the models. The polynomial spline-fitting algorithm approach we used is similar to the partial-rank correlation approach used to reduce the parameter space (Porco & Blower, 1998); however, a key advantage of approach is that it allows for interactions of parameters.

We analyzed models 0, 1, 2, and 3 as follows: the most influential parameters were allowed to vary; the remaining time-varying and famine related parameters were constrained at their null values, the starting populations of clusters 1 and 2 were set at 1,000, and the remaining non-time-varying parameters were constrained to the midpoint of the minimum and maximum values given in Table 1. For all four models, 7 parameters were allowed to vary; these 7 parameters for each model are given in Table 2. Thus, as all models have the same number of degrees of freedom, differing numbers of degrees of freedom cannot account for differences in model fit. The seven free parameters for each model were then optimized with respect to the discrepancy to determine which parameters produced the lowest discrepancies, using the Nelder–Mead downhill simplex method (Nelder & Mead, 1965) as implemented in the *optim* function in the base R statistical package. We then created 512 bootstrapped data sets for each model using a time-dependent parametric bootstrap, resampling the relative differences between the data and the optimum fit two data points at a time using *tdboot* from the *boot* package in R. Models were then fit to the bootstrap data sets in order to obtain 95% confidence intervals for the parameter estimates.

As a sensitivity analysis, we simulated single realizations of 1,024 parameter sets from a stochastic version of the null model with approximately equal populations. Then, we fit models 0, 1, 2, and 3 to these 1,024 parameter sets to determine the expected variation in model fit under the baseline model. Details are outlined in the Supplemental Information.

As an additional sensitivity analysis, we performed model fits with 10% of the population latently infected at the start of the epidemic. The fraction of latently infected individuals in the 1870s would have been very small, since genetic data indicates that *M. Tb* populations were small prior to expansion in the late 19th century: approximately 1–2 First Nations individuals in Western Canada had active TB disease prior to the 1870s (Pepperell et al., 2011a). In addition, endemic and epidemic forms of tuberculosis were not evident in to contemporary observers of these populations prior to the 1870s (Ferguson, 1928). We assume that when living conditions rapidly deteriorated in the 1870s and 1880s most or all of these latently infected individuals progressed to active disease, seeding the resulting epidemics on First Nations reserves. Thus for the main model, we began simulation with no latently infected individuals. However, this fraction is unknown. Therefore, as a sensitivity analysis, we performed fits with the 7 most influential parameters for models 0, 1, 2, and 3 with a larger starting latent fraction of 10%. Results are given below and graphical fits are given in Supplemental Information 2.

## Results

We identified the following parameters with the most important effects on disease dynamics, presented in order of importance, from most important to least important: the probability of fast progression (*p*), the effective contact rate (*β*), the starting fraction of the population with normal (lower) TB susceptibility at the start of the epidemic (*s*), the start time of the epidemic for cluster 1 (*t*_1_), the famine related change in the probability of fast progression (*δ_p_*), the start time of the epidemic for cluster 2 (*t*_2_), the protection conferred by latent infection to reinfection (*ζ*), the probability of developing infectious disease for those who develop active disease (*f*), and the TB death rate (*μ*_TB_). Thus, the final version of the famine model fit only incorporated one famine-related parameter: the famine related change in the probability of fast progression (*δ_p_*); the remaining famine-related parameters were constrained at their null values. For models 2 and 3, in addition to including the starting fraction of the population with normal (lower) TB susceptibility at the start of the epidemic (*s*), we included the relative increased susceptibility of the more susceptible group (*σ*) and the relative increased rate of progression for the fast progression group (*γ*), respectively, because the model reduces to the null model if these parameters are fixed at 1. So, these parameters must be not equal to 1 for the starting fraction with normal (lower) susceptibility to TB (*s*) to affect the model fit to the data.

**Figure 2:**
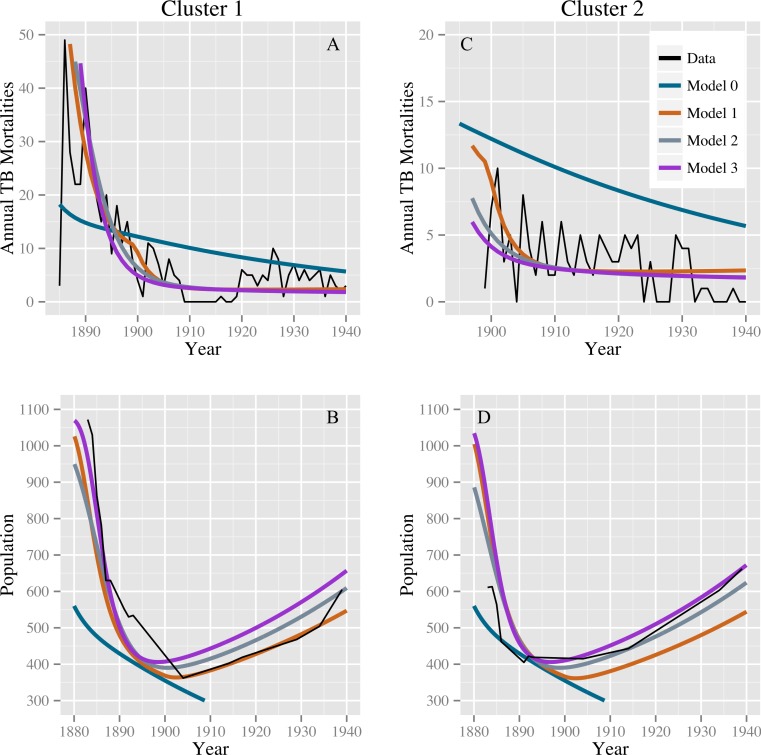
Model fits to the TB mortality time series and census data. Model fits to the TB mortality time series and census data for clusters 1 and 2 on the left (A and B, respectively) and right (C and D, respectively), respectively, and for models 0, 1, 2, and 3. Model 0 yields an implausibly poor fit to the TB mortality time series and census data, while models 1, 2, and 3 confer better fits, capturing the characteristic demographic decline and rise.

Table 2 shows the parameter estimates for models 0, 1, 2, and 3 with 95% confidence intervals. Models 1, 2, and 3 produce comparable discrepancies, while model 0 produces significantly higher discrepancies. Fits for models 0, 1, 2, and 3 to the TB mortality time series and census data are shown in Fig. 2.

Stochastic simulations under a null model failed to reproduce our observations. Models 0, 1, 2, and 3 all fit comparably well for stochastic data generated from the null model with roughly equal population sizes: we did not reproduce the observed preference for alternatives to model 0. For the 1,024 simulated data sets, the median ratios of the discrepancy of models 1, 2, and 3 compared with model 0 are 1.005 (IQR: 0.971, 1.074), 1.013 (IQR: 0.998, 1.082), and 1.017 (IQR: 0.998, 1.087), respectively. The stochastic simulations also failed to reproduce the observed population dynamics. For none of these simulations did the population start above 400 in 1883, fall below this level, and then recover to above 400—a trend clearly observed in the census data for both clusters. Using the observed data, the smallest ratio of discrepancy between fits for models 1, 2, and 3 and model 0 was 0.36 for the null model and model 3, indicating a strong preference for model 3. Out of 1,024 simulations, 4 simulated datasets had a ratio smaller than 0.36: one comparing models 0 and 1, two comparing models 0 and 2, and one comparing models 0 and 3. The four simulated datasets with a low discrepancy ratio comparing models 0 and 1 were not the same datasets that produced low discrepancy ratios when comparing models 0 and 2 and 0 and 3. However, in the fits of the observed data, the discrepancy for model 0 was high, whereas the discrepancies for models 1, 2, and 3 were all comparably lower. Based on these simulations, we conclude that stochastic variation is unlikely to account for model 0’s poor fit compared with models 1–3, and that model 0 is therefore not consistent with the observed data. Models 1, 2, and 3 (or combinations therein) are consistent with observed trends.

Lastly, a starting latent fraction of 10% did not appreciably affect results. Models 1–3 outperformed model 0. The discrepancies for models 0–3 are similar to those for fits with a starting latent fraction of 0% (Table 2) and are given by 202.6, 66.6, 65.0, and 63.0, respectively. Graphical results are shown in Supplemental Information 2.

## Discussion

**Figure 3:**
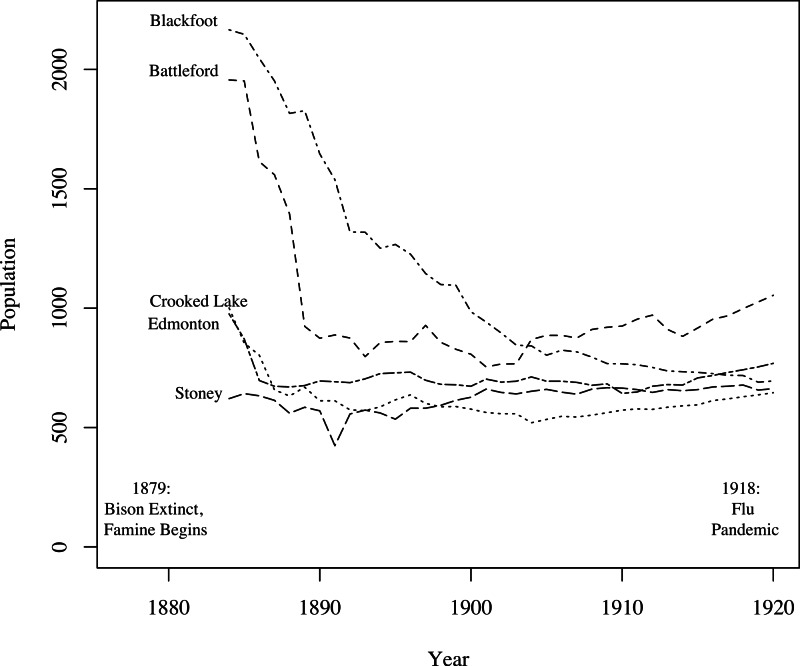
Graph of the populations of five groups of reserves (agencies) from 1884 to 1920 (Lux, 2001). For the census data for clusters 1 and 2, refer to Fig. 2. For four of the five agencies, populations decline following the onset of famine; most begin to recover around 1900.

A key challenge in fitting TB models to these data was fitting the mortality data while simultaneously producing reasonable population estimates. Figure 3, based on data from Lux (2001), shows population size trends in this region from 1884 to 1920 for five agencies (groups of reserves with shared administration, as opposed to individual reserves analyzed in this study). Clear trends are apparent: First, for four of the five agencies the population declined appreciably prior to 1900. The fifth, and smallest, may have declined in size prior to the onset of census data collection. Second, for four of the agencies the population did not continue to decline after 1900, and, in fact, began to grow again for three of the agencies. For the one agency for which the population continued to decline over the whole time period, the population did not fall below one-third of its 1884 value. The census data we have for clusters 1 and 2, shown in Fig. 2, are consistent with these trends, showing a sharp population decline following the start of TB epidemics followed by a period of increase after 1900. Thus, population trends observed in the 2 clusters for which time series mortality data were available are consistent with the 5 agencies shown here.

**Figure 4:**
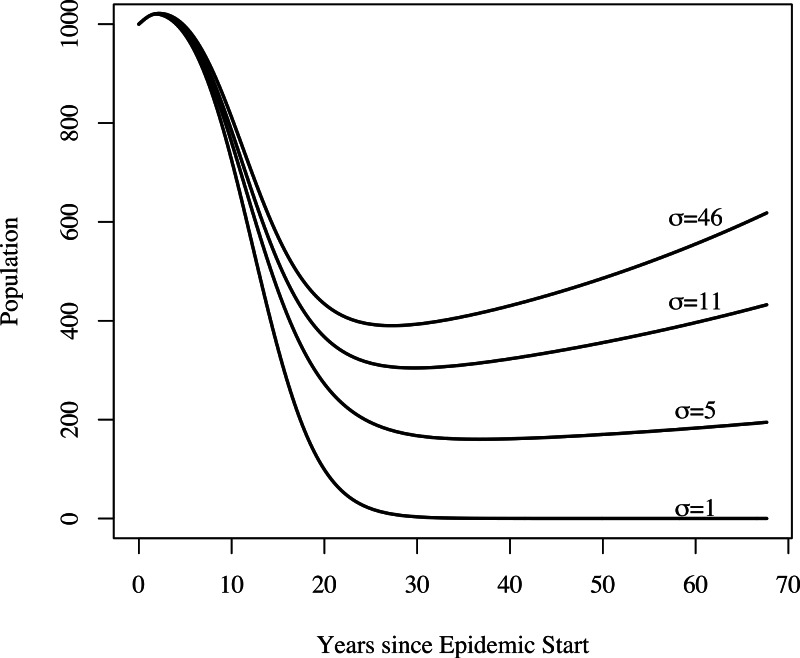
The effect of heterogeneity on population dynamics. Using the parameters from model 2’s best fit, as we decrease the relative increased susceptibility of the more susceptible group (*σ*) while keeping the mean susceptibility at the start of the epidemic constant, we see that the population decreases relative to model 2’s best fit. Greater heterogeneity allows us to reproduce the qualitative trend in population numbers of a sharp decrease in numbers followed by a slow increase.

In addition to yielding an implausibly poor fit to the TB mortality time series, model 0 failed to qualitatively reproduce the population dynamics. For model 0, populations associated with best fit to the mortality data decline and fail to recover. This is not the case for models 1, 2, and 3: we see a fall followed by a rise in the population levels consistent with both our census time series and regional trends shown in Fig. 3. Figure 4 shows the predicted population trends as the difference in relative susceptibility to infection (*σ*) increases, using parameters from the best fit of model 2. We can see here that more realistic demographic trends are achieved as we allow greater heterogeneity in susceptibility to infection.

**Figure 5:**
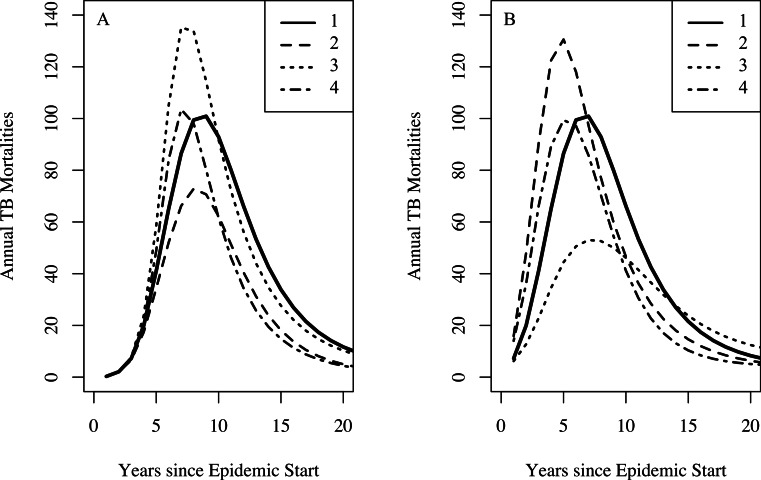
The effect of combinations of famine-related parameters on TB dynamics. Setting the effective contact rate (*β*) to 3.2, the relative increased susceptibility of the more susceptible group (*σ*) to 10, other famine-related parameters to their null values, and all remaining parameters to their midpoint values with no famine end time, we explore the dynamical effect of certain combinations of famine-related parameters on TB dynamics. For both graphs, curve 1 shows the TB mortality curve with no famine-related changes in parameters. (A) A three-fold increase in the background mortality (*δ*_*μ*_ = 3) rate leads to a decrease in the number of TB mortalities since death due to non-TB causes depletes those at-risk for TB death (curve 2). A three-fold decrease in immunity conferred by latency (*δ*_*ζ*_ = 0.3) results in more TB mortalities since more individuals become infected (curve 3). However, a three-fold increase in the background mortality rate in combination with a three-fold decrease in immunity conferred by latency results in a minimal change to the TB mortality curve (curve 4). (B) A 25% increase in the probability of fast-progression (*δ_p_* = 1.25) leads to an increase in the model-predicted number of TB mortalities during the first few years of the epidemic (curve 2). However, somewhat paradoxically, higher TB death rates do not necessarily lead to increased TB mortality at population-level. This is due to the fact that with lower TB death rates individuals with TB are infectious for more time and thus are able to infect more individuals, ultimately leading to a greater number of TB deaths in the population. With a two-fold increase in the TB death rate (*δ*_(*μ*_TB_)_ = 2), we see fewer TB mortalities (curve 3). However, a two-fold increase in the TB death rate in combination with a 25% increase in the probability of fast progression results in a minimal change to the TB mortality curve (curve 4).

For the famine model, we found a preference for a famine-related change in the probability of fast progression (*δ_p_*); however, the remaining famine related parameters did not appear to be as influential on dynamics of TB. There is abundant epidemiological evidence indicating that malnutrition increases both the background mortality rate (Bresnahan & Tanumihardjo, 2014) and the TB death rate (Rao et al., 1998; Gustafson et al., 2007). This lack of preference for famine-related changes to fit the observed data might be explained by the fact that certain combinations of famine-related changes in parameters are nearly dynamically neutral. Figure 5 shows that famine-related increases in risk of death (all causes) and death from TB could, in conjunction with other famine-related parameters, have only small effects on the predicted mortalities. These effects would not be possible to detect with the current data, particularly since other parameters, such as the epidemic start time, are allowed to vary.

Vynnycky & Fine (1999) explained the decline in TB in England and Wales in the late 18th and early 19th centuries with a declining effective contact rate over time and suggest that this may account for declines in other developed countries during the same time period. Here, introducing time-changing heterogeneity in TB susceptibility has similar dynamical effect: heterogeneity in TB susceptibility is dynamically equivalent to a time-decreasing average effective contact rate for this family of models. While alternate models that incorporate a declining effective contact rate may fit these data, we find the explanation of improved living conditions unsatisfactory. In order to explain our observed data, the increase in First Nations population densities associated with the shift from nomadic and semi-nomadic lifestyles to settlement on reserves would have had to immediately reverse itself. The late 19th and early 20th century were instead a time of increasing institutionalization of these populations (Lux, 2001; Waldram, Herring & Young, 2006); the expansion of the residential school system during this period is particularly notable (Milloy, 1999). The severe crowding, poor conditions and extensive TB transmission in schools of this period are well documented (Milloy, 1999). The effective contact rate is likely to have increased over this interval.

Based on our model fits, famine, genetic heterogeneity in susceptibility to infection, and genetic heterogeneity in risk of progression to active disease and combinations thereof are consistent with the observed demographic trends. While there may be other possible explanations, out of these three, we favor genetic heterogeneity in risk of progression to active disease for the following reasons. First, contemporary descriptions of the 19th/20th century epidemic noted that TB affected all families in these communities, and that half of the families were lost within a span of three generations (Ferguson, 1928). This suggests that the epidemic had the potential to impose very strong selection on existing genetic variation.

Second, previous work on these populations has shown that reserves that had early and more severe epidemics—the reserves studied here—currently have lower TB rates than do reserves that had later, less severe epidemics (Pepperell et al., 2011b). Our results could explain this modern-day difference in TB rates: in First Nations with early, severe epidemics, ecological drivers (chiefly famine) could have resulted in strong selection on existing genetic variation within these populations. We hypothesize that selection on populations with delayed TB epidemics was weaker as a result of effective TB treatments available during the later epidemics, and, possibly, less severe ecological drivers associated with later epidemics. There are marked disparities among contemporary First Nations with respect to their incidence of TB (Canadian Thoracic Society and the Public Health Agency of Canada, 2013). Genetic differences traceable to historical experiences with TB may account for some of these disparities.

Third and last, in the later years of the epidemic nearly all school-aged children were infected with *M.tb*, yet TB mortalities were considerably lower than in the early years of the epidemic. In several early 20th century surveys of children at school entry in this region all children examined showed signs of TB (Bryce, 1922; Milloy, 1999). Ferguson (1928) also noted universal infection of school-aged children in the latter years of the epidemic, providing evidence of recent infection. Since infection rates continued to be high at the end of the epidemic, this could indicate that the rate of progression to active disease had slowed, potentially due to a change in the population-averaged risk of progression due to genetic selection. Famine-related changes in the probability of fast progression are also consistent with these observations, but would not explain modern day differences between reserves with early and late epidemics.

In addition, selection for alleles that protect against infectious disease has been observed over short time scales, as we hypothesize occurred for TB in First Nations between 1883 and 1940. Increased frequencies of the *CCR*5Δ32 genotypes, which protect against HIV-1 infection and progression to AIDS, have been observed both in individuals at high risk for HIV-1 infection and in HIV-1 infected individuals at risk for progression to AIDS (Dean et al., 1996; Marmor et al., 2001; Lederman et al., 2006). In a study that recruited men who have sex with men in 1995 from various sites across the US, a higher prevalence of the *CCR*5Δ32 allele is significantly associated with increased age among Caucasian individuals from high-prevalence cities, but not from lower prevalence cities (Marmor et al., 2001). In addition, progression to AIDS is slower among individuals with either the heterozygous or homozygous genotype and thus, prior to the widespread availability of HIV-treatment, the frequency of both *CCR*5Δ32 genotypes was greatly elevated among individuals who had survived HIV-1 infection for more than 10 years (Dean et al., 1996). Further studies of genetic variation among First Nations Canadians would be required to reach any definitive conclusions on the role of human genetics and heterogeneity in susceptibility to TB in these populations.

There are several potential limitations to our approach. First, there are other potential explanations for the TB dynamics observed in this setting and Ferguson’s observation that half of all families were lost as a result of the epidemic. One such alternative explanation would be saturation of micro-networks (Veen, 1992; Meyers et al., 2005). However, given this extraordinarily high force of infection as apparent from the nearly universal infection of school-aged children, it is unlikely that the TB epidemic declined because of saturation of certain sub-populations (micro-networks) while others escaped the epidemic. In addition, saturation of micro-networks would fail to explain modern day differences between reserves with a history of early versus late epidemics (Pepperell et al., 2011b).

We did not account for epidemics of other diseases during this time period that could also have affected the observed demographic trends. Historical accounts indicate that intercurrent epidemics, while severe in some instances, were short-lived and that First Nations populations recovered quickly. For example, the 1918 influenza pandemic resulted in approximately 4,000 deaths total among all First Nations in Canada corresponding to a mortality rate of 37.7 per 1,000 population. For one First Nations group, for which detailed population level data were available, the population recovered to its pre-1918 levels within 5 years (Lux, 2001).

We limited our analysis to mortality data, the only available TB time series from this era, and census data. While we performed a rigorous process to determine which model parameters to fix and which to vary, it was not feasible to explore the full 16-dimensional parameter space for model 0, 22-dimensional parameter space for model 1, and 18-dimensional parameter spaces for models 2 and 3. In addition, we modeled the change in TB and population parameters due to improving famine conditions as a step-function rather than a gradual change. While crude, we believe this was the simplest way to include famine related factors in an absence of information allowing us to parameterize a more gradual change. Lastly, we did not include age structure (Brooks-Pollock, Cohen & Murray, 2010) since we did not have age-specific TB mortality data, age-specific background mortality data, and data on the population age-structure.

In conclusion, we explore the population-level effects of malnutrition and genetic heterogeneity in TB susceptibility on TB epidemics in First Nations reserves from 1885 to 1940. Malnutrition has previously been considered an individual-level risk factor and its population-level effects on TB epidemics have not previously been explored. This investigation demonstrates that changes in a population’s nutritional status can have significant effects on TB dynamics, while also producing the correct qualitative trends in population levels over this time period. In other settings, such famine-related changes in susceptibility may be necessary to explain TB dynamics. In addition, we find that the inclusion of heterogeneity in susceptibility to *M. tuberculosis* infection or risk of TB disease yields improved fits to both the TB mortality and census data while also producing the correct qualitative trends in population levels over this time period. Models that fail to account for famine or heterogeneity yield an implausibly poor fit to the TB mortality time series and fail to produce the correct trends in population size. In addition, modeling changes in genetic susceptibility over time has appreciable effects on population dynamics. Further research using human genetic data is required to conclusively determine the cause of modern-day disparities in these communities. In addition, disease dynamical effects of heterogeneity in susceptibility and fluctuating nutritional status should be investigated in other settings. Given the substantial dynamic effects observed with our models, development of TB control strategies aimed at improving nutrition, and/or targeted to highly susceptible individuals may yield substantial benefits at the population level.

## Supplemental Information

10.7717/peerj.1237/supp-1 Supplemental Information 1 Supplement to TB in First Nations

10.7717/peerj.1237/supp-2 Supplemental Information 2 TB Mortality and Census Data Raw data used in our analysis: TB Mortality and Census Data for 1883–1940.
