# Supplementary material for: Modeling historical tuberculosis epidemics among Canadian First Nations: effects of malnutrition and genetic variation
Source: PeerJ. 2015 Sep 24;3:e1237. doi: 10.7717/peerj.1237 (PMC4586807; doi:10.7717/peerj.1237)
Supplement: Supplemental Information 1 [file peerj-03-1237-s001.pdf]

Supplement to TB in Canadian First Nations at the turn-of-the twentieth century

S. F. Ackley, Fengchen Liu, Travis C. Porco, Caitlin S. Pepperell

Equations

Definitions

S, L, T_I, T_N, and R give the numbers of people in the susceptible, latent, infectious active disease, non-infectious active disease, and recovered groups, respectively, for the less susceptible group. Similarly, S', L', T'_I, T'_N, and R' give the numbers of people in the susceptible, latent, infectious active disease, non-infectious active disease, and recovered groups, respectively, for the more susceptible group. All parameters are given in table 1 of the main text or are subsequently defined. t refers to time and $H(t)$ refers to the Heaviside step function centered at $t = 0$.

Non-diseased total for less susceptible group:

$$N = S + L + R \tag{1}$$

Non-diseased total for more susceptible group:

$$N' = S' + L' + R' \tag{2}$$

Non-diseased total for both groups:

$$N_T = N + N' \tag{3}$$

Force of infection:

$$\lambda(t) = \frac{T_I + T'_I}{S + S' + L + L' + T_I + T'_I + T_N + T'_N + R + R'}\beta(t) \tag{4}$$

Effective contact rate:

$$\beta(t) = \beta(\delta_\beta)^{t-1880}H(t-1880) + \beta H(1880-t) \tag{5}$$

Probability of fast progression:

$$p(t) = p + H(\epsilon - t)(\delta_p - 1)p \tag{6}$$

Probability of fast progression for the more susceptible group:

$$p'(t) = \min\{p(t)\gamma, 1\} \tag{7}$$

TB death rate:

$$\mu_{TB}(t) = \mu_{TB} + H(\epsilon - t)(\delta_{\mu_{TB}} - 1)\mu_{TB} \tag{8}$$

Background death rate:

$$\mu(t) = \mu + H(\epsilon - t)(\delta_\mu - 1)\mu \tag{9}$$

Rate of progression from latency:

$$\nu(t) = \nu + H(\epsilon - t)(\delta_\nu - 1)\nu \tag{10}$$

Protective immunity:

$$\zeta(t) = \zeta + H(\epsilon - t)(\delta_\zeta - 1)\zeta \tag{11}$$

Births per year, where Λ is the birthrate per 1000:

$$\Lambda(t) = (\Lambda + H(\epsilon - t)(\delta_\Lambda - 1)\Lambda)\frac{N + N'}{1000} \tag{12}$$

Differential Equations

Susceptible individuals, less susceptible group:

$$\frac{dS}{dt} = -\mu S - \lambda(t)S + \Lambda(t)\frac{N}{N_T} \tag{13}$$

Latently infected individuals, less susceptible group:

$$\frac{dL}{dt} = -\mu L + (1 - p(t))\lambda(t)S - v(t)L - p(t)(1 - \zeta(t))\lambda(t)L \tag{14}$$

Infectious active TB cases, less susceptible group:

$$\begin{aligned}\frac{dT_I}{dt} = &- (\mu + \mu_{TB})T_I + f[p(t)\lambda(t)S + v(t)L \\ &+ p(t)(1 - \zeta(t))\lambda(t)L + \omega R + p(t)(1 - \zeta(t))\lambda(t)R] - cT_I\end{aligned} \tag{15}$$

Non-infectious active TB cases, less susceptible group:

$$\begin{aligned}\frac{dT_N}{dt} =& -(\mu + \mu_{TB})T_N + (1-f)[p(t)\lambda(t)S + v(t)L \\ &+ p(t)(1-\zeta(t))\lambda(t)L + \omega R + p(t)(1-\zeta(t))\lambda(t)R] - cT_N \end{aligned} \tag{16}$$

Recovered individuals, less susceptible group:

$$\frac{dR}{dt} = -\mu R - \omega R + c(T_I + T_N) - p(t)(1-\zeta(t))\lambda(t)R \tag{17}$$

Susceptible individualss, more susceptible group:

$$\frac{dS'}{dt} = -\mu S' - \sigma\lambda(t)S' + \Lambda(t)\frac{N'}{N_T} \tag{18}$$

Latently infected individuals, more susceptible group:

$$\frac{dL'}{dt} = -\mu L' + (1-p'(t))\sigma\lambda(t)S' - v(t)L' - p'(t)(1-\zeta(t))\sigma\lambda(t)L' \tag{19}$$

Infectious active TB cases, more susceptible group:

$$\begin{aligned}\frac{dT_I'}{dt} =& -(\mu + \mu_{TB})T_I' + f[p'(t)\sigma\lambda(t)S' + v(t)\gamma L' + p'(t)(1-\zeta(t))\sigma\lambda(t)L' \\ &+ \omega R' + p'(t)\sigma(1-\zeta(t))\lambda(t)R'] - cT_I' \end{aligned} \tag{20}$$

Non-infectious active TB cases, more susceptible group:

$$\begin{aligned}\frac{dT_N'}{dt} =& -(\mu + \mu_{TB})T_N' + (1-f)[p'(t)\sigma\lambda(t)S' + v(t)\gamma L' + p'(t)\sigma(1-\zeta(t))\lambda(t)L' \\ &+ \omega R' + p'(t)\sigma(1-\zeta(t))\lambda(t)R'] - cT_N' \end{aligned} \tag{21}$$

Recovered Individuals, more susceptible group:

$$\frac{dR'}{dt} = -\mu R' - \omega R' + c(T_I' + T_N') - p'(t)\sigma(1-\zeta(t))\lambda(t)R' \tag{22}$$

Discrepancy Function on the Relative Scale

$$D = \sum_{i=1}^{100}(\log(m_i+1) - \log(m_i'+1))^2 + \frac{100}{26}\sum_{i=1}^{26}(\log(p_i+1) - \log(p_i'+1))^2 \tag{23}$$

Where D is the total discrepancy, m_i is an observed mortality data point and m_i' is the corresponding simulated mortality data point, and p_i is an observed population data point and p_i' is the corresponding simulated population data point. The population and mortality

discrepancies are weighted equally, so the weight $\frac{100}{26}$ is applied to the population data to compensate for the fact that there are fewer population points (26 population data points versus 100 mortality data points).

Below we show the distributions of bootstrapped discrepancies for each of the models.

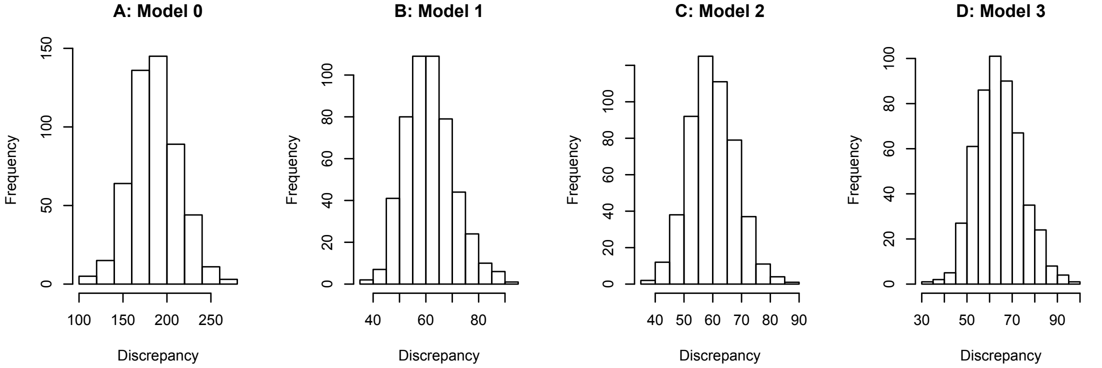

Fits with Discrepancy on the Additive Scale

We used the relative scale for the main analysis to allow us to perform a bootstrap to obtain confidence intervals. Here we show the fits on the additive scale using the following discrepancy function:

$$D = \sum_{i=1}^{100}(m_i - m_i')^2 + 0.01\sum_{i=1}^{26}(p_i - p_i')^2 \tag{24}$$

The population data was down-weigthed since the populations are an order of magnitude greater than the mortalities. Other weights yield similar results. The discrepancies for models 0-3 are given by: 14,491, 6,549, 6,379, and 7,953, respectively.

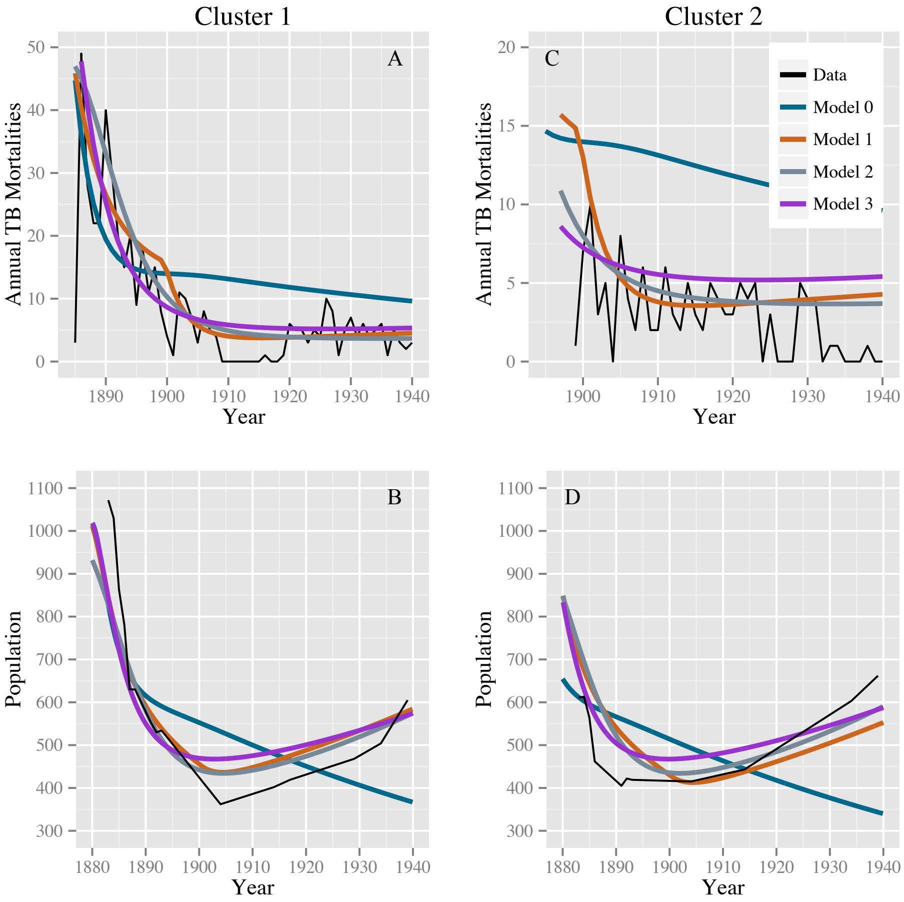

Fits with 10% of the Starting Population Latently Infected

Using the discrepancy function on the relative scale, we fit models 0, 1, 2, and 3 with 10% of the starting population latently infected. We consider 10% a reasonable upper bound for the fraction latently infected. Below we show the corresponding fits; we find that starting with a greater fraction of latents does not substantively affect the result that model 0 fails to replicate the observed trends, while models 1, 2, and 3 do. Models 0, 1, 2, and 3 yield the following discrepancies, respectively: 202.6, 66.6, 65.0, and 63.0.

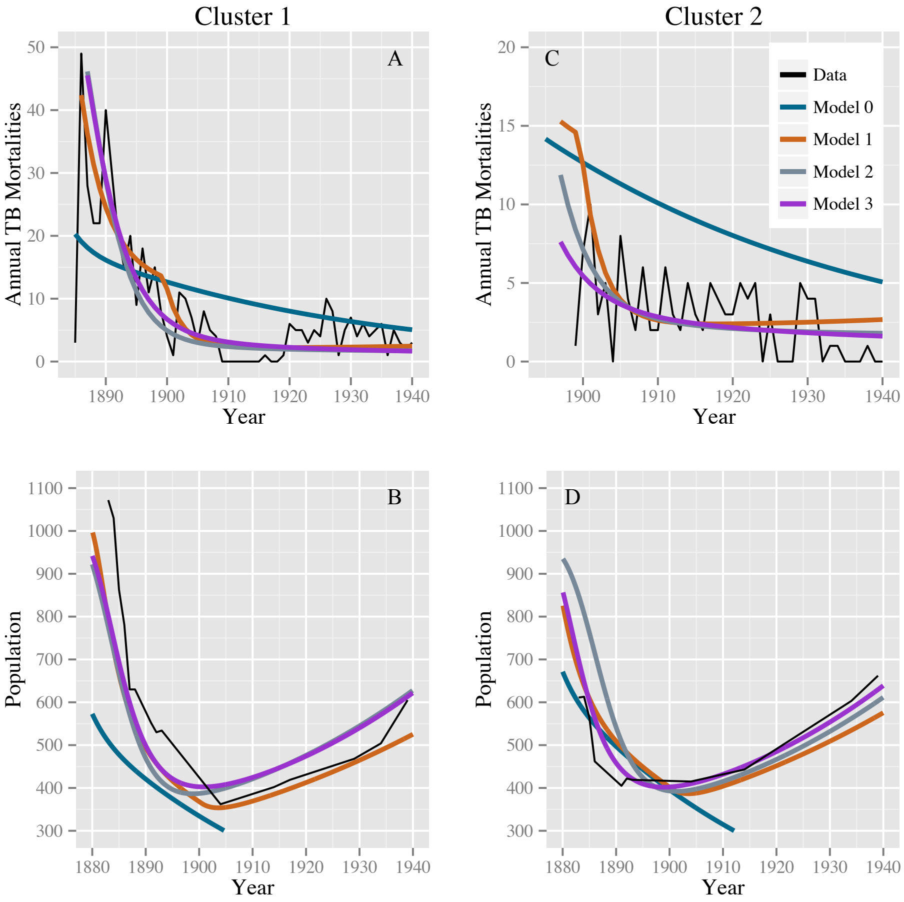

Sensitivity Analyses

We simulated 1024 single realizations of a TB epidemic for clusters 1 and 2 using a stochastic version of model 0, the null model. To do this, differential equations were converted to stochastic difference equations. At each time step, each individual could stay in their current state or transition to a new state using binomial transition probabilities based on the rates of transition outlined in figure 1 and a time step of 1/120 year. Parameter ranges were taken from table 1 of the main text. Populations for clusters 1 and 2 were constrained to start between 800 and 1200 individuals since, based on the fact the two clusters bottomed out at approximately same level and rose to approximately same level along parallel trajectories, we assume that populations started off at approximately equal levels as well.

First, 1024 parameter set were obtained by sampling from a uniform distribution of the parameter ranges. Then, for each parameter set, the stochastic simulation data was

generated for both clusters 1 and 2. Then, this simulated data was fit to the final models for models 0-3 described in the results section using a discrepancy on the relative scale. Results are given in the main text.
